# Despite COVID-19 Member States Need to Adequately Resource WHO’s Work to Address Alcohol Harm

**DOI:** 10.34172/ijhpm.2020.234

**Published:** 2020-11-23

**Authors:** Øystein Bakke, Eva Sigrid Braaten, Sally Casswell

**Affiliations:** ^1^Global Alcohol Policy Alliance, Auckland, New Zealand.; ^2^FORUT, Gjøvik, Norway.; ^3^SHORE & Whariki Research Centre, Massey University, Auckland, New Zealand.

## Dear Editor,

 The crisis imposed by the coronavirus disease 2019 (COVID-19) will challenge the World Health Organization (WHO) for some time to come. However, the COVID-19 pandemic has illustrated the importance of a collaborative global response when faced with a global risk to health and the value of an intergovernmental agency on health. Despite the pressures on WHO and its Member States maintaining and strengthening a collaborative global response to risk factors for health is essential.

## Alcohol – an Increasing Global Burden

 Alcohol ranks high on the list of risk factors for the Global Burden of Disease. Globally, in 2016, 5.1% of all disability adjusted life years were attributable to alcohol use, with almost 3 million deaths.^1^ Alcohol is a risk factor for communicable and non-communicable diseases, injuries, and mental health problems. Socio-economic harm, which can be substantial, is not factored in these calculations. Since 1990 global per capita alcohol use has increased substantially (from 5.9 litres to 6.5 litres in 2017^2^), with the highest growth occurring in many middle-income countries. Unless effective government policies are put in place this trend is forecast to continue with increasing impact on public health.^2^ The global community has already set a goal to reduce alcohol harm, in the Sustainable Development Goals target 3.5. However, there is a growing consensus that the global public health community is failing to address the burden of alcohol.^3,4^

## Urgent Need for Realistic Funding

 A focus on alcohol at the 72nd World Health Assembly in May 2019 led to a request to WHO Director-General, Dr. Tedros, to “adequately resource the work on the harmful use of alcohol.”^5^ Current funding levels are remarkably small at global, regional, and country levels. Only an estimated US$1 million per year^[1]^ was allocated for the WHO Head Quarter efforts to develop capacity, instruments, and technical advice for the implementation of the global strategy to reduce the harmful use of alcohol. In contrast the assessed funding for the implementation of the Framework Convention on Tobacco Control in 2018-2019 was about US$8.8 million.^7^ The commitment to provide adequate resources is clearly crucial to any realistic hope for a limitation of alcohol harm.

 The global strategy to reduce the harmful use of alcohol was endorsed by the 63rd World Health Assembly in 2010. It outlines cost-effective interventions to reduce the health burden from alcohol. Implementation of the global strategy has, however, been slow.^3,8^ This, undoubtedly, is a consequence of conflict of interest, industry influence and lack of political will both globally and nationally as well as lack of resources. There is a particular need to support low- and middle-income countries where alcohol control policy measures are less likely to be in place and alcohol consumption is on the rise. With the projected increase in disease burden and the agreement to develop a global action plan to implement the global strategy,^5^ the time is right for adequate funding to be allocated to enhance technical skills and advocacy for effective policies. Even before the action plan is in place, the most cost-effective interventions are being promoted by the WHO-led SAFER initiative – aimed at providing support for Member States in reducing the harmful use of alcohol. Funding is urgently needed to implement this initiative.^9^

## Lack of Philanthropic and Earmarked Support

 Much of WHO funding is now in the form of earmarked funds either from Member States or philanthropic organisations. In the 2018-2019 programme period less than one fifth of the total WHO budget was in the form of unallocated ‘assessed funding.’^10^However, most (60%) of the funding for alcohol (as part of the alcohol, drugs and addictive behaviour output area) was from assessed funding; a further 22% was from Voluntary Specified Contributions from Member States and the rest from other minor funding streams. Compared with other risk factors, philanthropic foundations have failed to be major sources of funding for WHO’s alcohol activities. The philanthropic funding of WHO’s work on alcohol, drugs and addictive behaviours in 2018-2019 totalled US$15 000, mostly through the South East Asia Regional Office,^6^ while, in the same budget period, the Bloomberg Family Foundation provided US$22 million to WHO for prevention of non-communicable diseases, violence and injuries.^10^

 Just as there are no funders coming forward to support the alcohol portfolio in WHO, there are few funders (government or private) willing to support civil society efforts to address alcohol harm, either for community programs or for policy advocacy at the national or global levels.^11^

 As illustrated by the figures above, compared to other public health challenges alcohol is severely under-funded. Funding commensurate with the health burden is needed in order to fulfil the ambition of accelerating action. Despite the difficulties imposed by the COVID-19 pandemic, the need for a much-accelerated response to alcohol harm is urgent. This will not happen unless Member States provide funding proportional to the health burden from alcohol.

## Ethical issues

 Not applicable.

## Competing interests

 Authors declare that they have no competing interests.

## Authors’ contributions

 ØB and SC were responsible for the conception and design of the manuscript. ØB obtained the data and drafted the manuscript. ØB, ESB, and SC critically revised and made important intellectual contributions to the manuscript.

## Endnotes

 [1] The projected budget for the ‘alcohol, drugs and addictive behaviour’ output area for 2018-2019 was US$8.8 million with 6.2 million actually allocated (5.9 million spent).^6^ A rough estimate is that alcohol makes up at most two-thirds of the expenditure, less than US$4 million for the two-year period on alcohol covering global, regional and country level work – and 1 million per year for the WHO Head Quarter activities.

## Authors’ affiliations

^1 ^Global Alcohol Policy Alliance, Auckland, New Zealand. ^2^FORUT, Gjøvik, Norway. ^3^SHORE & Whariki Research Centre, Massey University, Auckland, New Zealand.
